# Efficacy of Hyaluronan‐Enriched Transfer Medium in Frozen–Thawed Embryo Transfer: A Retrospective Single‐Center University Hospital Study Using a Historical Cohort

**DOI:** 10.1002/rmb2.70074

**Published:** 2026-09-11

**Authors:** Shoko Toyoda, Seung Chik Jwa, Suguru Kamishiro, Yumi Sato, Yoko Fujimoto, Keiko Kagawa, Mizuho Sugiyama, Mai Ohashi, Hiroyuki Fujiwara

**Affiliations:** ^1^ Department of Obstetrics and Gynecology Jichi Medical University Tochigi Japan

**Keywords:** clinical pregnancy, frozen–thawed embryo transfer, hyaluronan, hyaluronan‐enriched transfer medium, recurrent implantation failure

## Abstract

**Objective:**

Since 2023, our university hospital has incorporated hyaluronan‐enriched transfer medium (HETM) into all frozen–thawed embryo transfers (FETs). This study aimed to evaluate whether HETM improved clinical pregnancy rates in FET, particularly in patients with recurrent implantation failure (RIF).

**Methods:**

We analyzed 368 FET cycles using a conventional hyaluronan‐free transfer medium and 358 FET cycles using HETM, in which thawed embryos were incubated for 2–4 h before transfer. The main outcome was clinical pregnancy. Multiple logistic regression was used to evaluate the effectiveness of HETM.

**Results:**

The mean age of patients was 37.4 years (standard deviation = 4.2), and RIF, defined as failure to achieve pregnancy after two or more previous embryo transfers, accounted for 51.2%. Clinical pregnancy rates were significantly higher in the HETM group (37.7%) than in the control group (27.2%) (*p* = 0.002). Stratified analyses showed that HETM was associated with higher clinical pregnancy rates in RIF cycles (adjusted odds ratio = 2.4 [95% confidence interval: 1.4–3.9]). An additional analysis restricted to patients with three or more previous failed embryo transfers showed similar results.

**Conclusion:**

This study demonstrates HETM use is associated with improved clinical pregnancy rates in FET, particularly in cases of RIF.

## 1 Introduction

Hyaluronan (HA) is a naturally occurring glycosaminoglycan that is abundant in human follicular fluid and the endometrium, where it plays a critical role in embryo implantation [1]. Through binding to its receptor CD44, which is expressed on the embryo surface from the oocyte to the blastocyst stage and on the endometrial stroma—with peak expression during the implantation window [2]—HA facilitates cell–cell and cell–matrix adhesion. In mice, HA biosynthesis increases dramatically at the time of embryo implantation and decreases to basal levels the following day, suggesting its physiological importance in the implantation process [3]. In addition, the hydrodynamic properties of HA, including tissue hydration and expansion effects, create a favorable environment for embryo migration and attachment.

Based on these mechanisms, hyaluronan‐enriched transfer medium (HETM) has been developed to improve embryo implantation rates in assisted reproductive technology (ART). Several randomized controlled trials (RCTs) have demonstrated that HETM can significantly improve clinical pregnancy and implantation rates in fresh embryo transfers [4, 5]. A systematic review published in 2020 that analyzed RCTs showed overall effectiveness [6]. More recent studies have reported particular benefits in specific patient populations, including patients with recurrent implantation failure (RIF) [7], and in transfers involving poor‐quality embryos [8].

However, the efficacy of HETM in frozen–thawed embryo transfer (FET) remains controversial. While several studies have reported improved clinical pregnancy rates with HETM use in FET cycles [4, 7, 8, 9], other reports have found no significant differences in pregnancy outcomes [10, 11, 12]. These inconsistent results may be attributable not only to differences in study populations—such as varying proportions of patients with recurrent implantation failure (RIF), embryo quality, and embryo developmental stage—but also to methodological differences across studies, including the composition of the control medium, as some studies used media containing low concentrations of hyaluronan rather than completely HA‐free media [11, 12]. Given the increasing prevalence of FET in contemporary ART practice and the limited evidence specifically addressing HETM efficacy in this setting, further investigation is warranted.

In February 2023, our university hospital implemented HETM for all FET procedures. This single‐center retrospective study aimed to evaluate the effectiveness of HETM by comparing clinical outcomes in the year before and after its introduction, using an HA‐free transfer medium as the control. Because all FET cycles performed during the study periods were included, this study also enabled us to examine the potential effects of HETM across a broad range of clinical factors, including embryo quality, embryo developmental stage, and the number of previous embryo transfers, with particular attention to patient subgroups including those with RIF.

## 2 Materials and Methods

**FIGURE 1:**
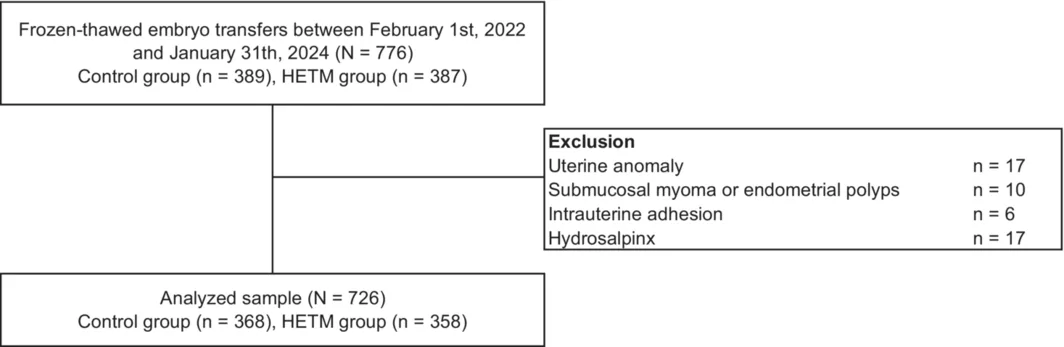
Sample flow chart.

This was a retrospective study based on a medical chart review at Jichi Medical University Hospital. Patients who underwent FET between February 2022 and January 2024 were eligible. A total of 776 FET cycles were performed during the study period, including 387 cycles using HETM (HETM group; February 2023–January 2024) and 389 cycles (control group; February 2022–January 2023). Cycles complicated by uterine anomalies (*n* = 17), submucosal myoma or endometrial polyps (*n* = 10), intrauterine adhesions (*n* = 6), or hydrosalpinx (*n* = 17) were excluded. Finally, 358 FET cycles (222 patients) in the HETM group and 368 FET cycles (213 patients) in the control group were included in the analysis (Figure 1). The study was approved by the Institutional Review Board of Jichi Medical University Hospital (approval no. 23–205).

### 2.1 Endometrial Preparation Protocols for Frozen–Thawed Embryo Transfer Cycles

During the study period, more than 90% of FET cycles were conducted using hormone replacement cycles (HRC) (Table 1). Estradiol transdermal patches (Estrana Tape 0.72 mg, Hisamitsu Pharmaceutical Co., Japan) were started within 4 days of the onset of menstruation at a fixed dose (four sheets every other day) for 10–14 days to promote endometrial proliferation and inhibit spontaneous follicle growth. When endometrial thickness was ≥ 7 mm on transvaginal ultrasound with adequate serum estradiol (E2) levels and without elevated serum progesterone (P4) levels, progesterone vaginal tablets (Lutinus, Ferring Pharmaceuticals, Japan; or Utrogestan, FujiPharma, Japan; or Luteum, ASKA, Japan) were administered at the manufacturer‐recommended doses, and the date of FET was determined according to the embryo stage at transfer. Estradiol supplementation and luteal support were continued until pregnancy confirmation. When pregnancy was confirmed, estradiol transdermal patches and progesterone vaginal tablets were continued until 9 weeks' gestation.

**TABLE 1:** Characteristics and treatment information stratified by HETM (*n* = 726).

Variables	Control group (*n* = 368)	HETM group (*n* = 358)	*p* ^a^
Age (years)	37.6 (4.4)	37.1 (4.1)	0.18
Husband age	38.6 (5.7)	38.9 (5.6)	0.55
BMI	23.0 (3.6)	23.3 (3.7)	0.49
Gravidity			
0	137 (37.2%)	134 (37.4%)	0.96
1	231 (62.8%)	224 (62.6%)	
Parity			
0	238 (64.7%)	235 (65.6%)	0.78
1	130 (35.3%)	123 (34.4%)	
Infertility diagnosis			
Tubal factor	53 (14.4%)	35 (9.8%)	0.06
Male factor	33 (9.0%)	50 (14.0%)	0.03
Endometriosis	57 (15.5%)	55 (15.4%)	0.96
Antisperm antibody	3 (0.8%)	2 (0.6%)	1.00
Fertility preservation for cancer patients	0 (0%)	2 (0.6%)	0.24
PCOS	6 (1.6%)	28 (7.8%)	< 0.001
Unexplained	218 (59.2%)	180 (50.3%)	0.02
Number of previous embryo transfers			
0	100 (27.2%)	109 (30.5%)	0.51
1	72 (19.6%)	73 (20.4%)	
RIF^b^	196 (53.3%)	176 (49.2%)	

*Note:* Data are presented as mean (SD) for continuous variables and *n* (%) for dichotomous variables.

Abbreviations: BMI, body mass index; HETM, hyaluronan‐enriched transfer medium; PCOS, polycystic ovary syndrome; RIF, recurrent implantation failure.

^a^ *p* values were assessed using chi‐squared or Student's *t*‐test.

^b^ Defined as patients without pregnancy after two or more previous embryo transfers.

In the remaining FET cycles, endometrial preparation was performed either in an ovulation induction cycle or a natural cycle. In both protocols, follicular development was confirmed by ultrasound examination. For ovulation induction, ovulation was induced using clomiphene citrate (CC) or letrozole, according to the treating physician's discretion. For natural‐cycle FET, ovulation was identified based on both ultrasonographic confirmation of follicular development and detection of the luteinizing hormone (LH) surge; the day after the LH surge was regarded as the day of ovulation, and embryo transfer was scheduled according to the embryo stage at transfer. Alternatively, ovulation was triggered with recombinant hCG after ultrasound confirmation of follicular development, and embryo transfer was performed according to the embryo stage at transfer.

### 2.2 Preparation of HETM and Embryo Transfer

For the transfer medium, 0.8 mL of HETM (EmbryoNida, KITAZATO Corporation, Shizuoka, Japan) was used in the HETM group, whereas Sydney IVF Blastocyst Medium (COOK Medical Japan G.K., Tokyo, Japan), which contains no HA, was used in the control group. Both medium were placed into embryo transfer dishes and incubated at 37°C in an atmosphere containing 6.0% CO_2_ and 5.0% O_2_ for ≥ 12 h for equilibration on the day before FET. On the day of FET, following thawing and assisted hatching (AHA), the embryo was cultured for 2–4 h in EmbryoNida in the HETM group and in Sydney IVF Blastocyst Medium in the control group. After post‐thaw culture and morphological grading, embryos were transferred into the patient's uterus using a 4.7 Fr embryo transfer catheter (KITAZATO Corporation, Shizuoka, Japan) under transabdominal or transvaginal ultrasound guidance. The loading medium was EmbryoNida in the HETM group and Sydney IVF Blastocyst Medium in the control group. For embryo loading into the catheter, three 5‐μL air bubbles and two 5‐μL volumes of loading medium were alternately aspirated into the embryo transfer catheter. The embryo was then aspirated together with the medium immediately proximal to the air bubble at the catheter tip. Thus, the main procedural difference between the two groups was the transfer medium used during pre‐transfer embryo incubation, with HETM used after February 2023 and a conventional HA‐free medium used before its introduction.

### 2.3 Main Outcome Measures

The main outcome of this study was clinical pregnancy, defined as confirmation of an intrauterine gestational sac. Secondary outcomes were implantation and miscarriage. Implantation was defined as an elevation in serum hCG at 4 weeks' gestation. Miscarriage was defined as complete expulsion of products of conception.

### 2.4 Covariates

Patient characteristics (age, body mass index [BMI], parity, and infertility diagnosis) and treatment information (number of previous embryo transfers, endometrial preparation protocol, number of embryos transferred, embryo stage at transfer [blastocyst or cleavage stage], and embryo quality [good or poor]) were extracted from the electronic medical records. In this study, because information on embryo quality in prior embryo transfer cycles was unavailable in our data set, RIF was operationally defined as failure to achieve pregnancy after two or more previous embryo transfers. To further examine the robustness of the findings, an additional conservative analysis was performed in patients with three or more previous failed embryo transfers. At our institution, add‐on interventions for selected patients with RIF, including endometrial scratching and ERA/EMMA/ALICE assays (Igenomix, Spain), were not performed during the control period. During the HETM period, endometrial scratching‐related procedures were performed in 32 cases, and ERA/EMMA/ALICE assays was performed in 5 cases. Relative to the 358 FET cycles in the HETM period, these frequencies were approximately 8.9% and 1.4%, respectively. Thus, although these interventions were infrequently used, temporal differences in the use of add‐on interventions could not be completely excluded. In addition, hysteroscopy was routinely performed as a screening examination before the first embryo transfer in all patients throughout the study period. In addition, hysteroscopy was routinely performed as a screening examination before the first embryo transfer in all patients throughout the study period. Good‐quality embryos were defined as Gardner grade ≥ BB for blastocyst transfers and Veeck grade ≥ 2 for cleavage‐stage embryo transfers. Poor‐quality embryos were defined as embryos not meeting these criteria. No preimplantation genetic testing for aneuploidy (PGT‐A) cycles were included in this study, because PGT‐A was not performed at our institution during the study period.

### 2.5 Statistical Analysis

First, we evaluated whether patient characteristics and treatment factors differed between the HETM and control groups. Categorical variables were compared using the chi‐square test, and continuous variables were compared using Student's *t*‐test. Second, clinical pregnancy, implantation, and miscarriage rates were compared between the HETM and control groups. Third, associations between HETM use and clinical outcomes were examined using logistic regression, and crude and adjusted odds ratios (ORs) with 95% confidence intervals (CIs) were calculated. Covariates included in the adjusted models were maternal age, BMI, parity, infertility diagnosis, number of embryos transferred, and embryo quality. We further stratified analyses according to the number of previous embryo transfer attempts (0, 1, ≥ 2, and ≥ 3). Finally, subgroup analyses were conducted for blastocyst transfers (*n* = 653), cleavage‐stage transfers (*n* = 73), transfers of good‐quality embryos (*n* = 390), and transfers of poor‐quality embryos (*n* = 336). All analyses were performed using Stata/MP version 18.1 (StataCorp, College Station, TX, USA). A two‐tailed *p* < 0.05 was considered statistically significant.

## 3 Results

### 3.1 Baseline Characteristics

Baseline characteristics are shown in Tables 1 and 2. The mean maternal age was 37.4 years (SD = 4.2) with no significant difference between groups (HETM group: 37.1 years vs. control group: 37.6 years, *p* = 0.18). Most patients were nulliparous (approximately 65% in both groups, *p* = 0.78). There were some differences in infertility diagnosis, with higher proportions of male factor (14.0% vs. 9.0%, *p* = 0.03) and polycystic ovary syndrome (PCOS) (7.8% vs. 1.6%, *p* < 0.001) in the HETM group. RIF cases accounted for approximately half of the cohort in both groups (49.2% vs. 53.3%, *p* = 0.51).

**TABLE 2:** Treatment information and pregnancy outcomes stratified by HETM.

Variables	Control group (*n* = 368)	HETM group (*n* = 358)	*p* ^a^
Number of embryo transferred			
1	258 (70.1%)	293 (81.8%)	< 0.001
2	110 (29.9%)	65 (18.2%)	
Embryo stage at transfer			
Early cleavage	44 (12.0%)	29 (8.1%)	0.08
Blastocyst	324 (88.0%)	329 (91.9%)	
Embryo quality			
Good quality embryos^b^	180 (48.9%)	210 (58.7%)	0.01
Poor quality embryos^c^	188 (51.1%)	148 (41.3%)	
Endometrial preparation protocol			
HRC	343 (93.2%)	339 (94.7%)	0.66
Ovulation induction	22 (6.0%)	16 (4.5%)	
Natural	3 (0.8%)	3 (0.8%)	

Abbreviations: HETM, hyaluronan‐enriched transfer medium; HRC, hormone replacement cycle.

^a^ *p* values were calculated using the chi‐squared test for the overall comparison of each grouped variable between the control and HETM groups, not for each individual category. Therefore, the *p*‐value cells were merged across the corresponding categories.

^b^ Good quality embryo includes cleavage embryos that were graded as 2 according to the Veeck classification system and blastocysts that were graded as BB according to the Gardner classification system.

^c^ Poor‐quality embryos were defined as embryos not meeting the criteria for good‐quality embryos.

### 3.2 Treatment Characteristics

Regarding treatment characteristics, single embryo transfer was more common in the HETM group (81.8% vs. 70.1%, *p* < 0.001), and good‐quality embryos were transferred more frequently (58.7% vs. 48.9%, *p* = 0.01) while poor‐quality embryos were transferred less frequently in the HETM group than in the control group (41.3% vs. 51.1%, *p* = 0.01). The majority of cycles used hormone replacement cycles for endometrial preparation (94.7% vs. 93.2%, *p* = 0.66).

### 3.3 Clinical Outcomes

Crude and adjusted odds ratios of HETM for clinical outcomes are shown in Table 3. The clinical pregnancy rate was significantly higher in the HETM group (37.7%, 135/358) than in the control group (27.2%, 100/368) (*p* = 0.002). After adjusting for confounders, the adjusted OR for clinical pregnancy was 1.7 (95% CI: 1.2–2.3; *p* = 0.003). Similarly, implantation was significantly higher with HETM use (53.4% vs. 41.0%; adjusted OR: 1.6, 95% CI: 1.2–2.2; *p* = 0.002). The miscarriage rate tended to be lower in the HETM group (23.7% vs. 34.0%), although this did not reach statistical significance (adjusted OR: 0.6, 95% CI: 0.3–1.0; *p* = 0.09).

**TABLE 3:** Crude and adjusted odds ratio of HETM for clinical outcomes.

Variables	Control group	HETM group	*p*	Crude OR (95% CI)	*p*	Adjusted OR (95% CI)^a^	*p*
All (*n* = 726)	(*n* = 368)	(*n* = 358)					
Implantation	151 (41.0%)	191 (53.4%)	0.001	**1.6 (1.2 to 2.2)**	**0.001**	**1.6 (1.2 to 2.2)**	**0.002**
Clinical pregnancy	100 (27.2%)	135 (37.7%)	0.002	**1.6 (1.2 to 2.2)**	**0.003**	**1.7 (1.2 to 2.3)**	**0.003**
Miscarriage^b^	34 (34.0%)	32 (23.7%)	0.08	0.6 (0.3 to 1.1)	0.084	0.6 (0.3 to 1.0)	0.09
No. of previous ETs = 0 (*n* = 209)	(*n* = 100)	(*n* = 109)					
Implantation	44 (44.0%)	70 (64.2%)	0.003	**2.3 (1.3 to 4.0)**	**0.004**	**2.7 (1.4 to 5.0)**	**0.002**
Clinical pregnancy	35 (35.0%)	48 (44.0%)	0.18	1.5 (0.8 to 2.6)	0.18	**1.9 (1.0 to 3.7)**	**0.043**
Miscarriage^b^	13 (37.1%)	10 (20.8%)	0.10	0.4 (0.2 to 1.2)	0.11	0.4 (0.1 to 1.3)	0.14
No. of previous ETs = 1 (*n* = 145)	(*n* = 72)	(*n* = 73)					
Implantation	33 (45.8%)	38 (52.1%)	0.45	1.3 (0.7 to 2.5)	0.45	1.1 (0.5 to 2.2)	0.82
Clinical pregnancy	25 (34.7%)	27 (37.0%)	0.78	1.1 (0.6 to 2.2)	0.78	0.9 (0.4 to 2.0)	0.86
Miscarriage^b^	6 (24.0%)	8 (29.6%)	0.65	1.3 (0.4 to 4.6)	0.65	1.1 (0.2 to 6.8)	0.91
No. of previous ETs ≥ 2 (*n* = 372):RIF^c^	(*n* = 196)	(*n* = 176)					
Implantation	74 (37.8%)	83 (47.2%)	0.067	1.5 (1.0 to 2.2)	0.067	**1.6 (1.0 to 2.5)**	**0.04**
Clinical pregnancy	40 (20.4%)	60 (34.1%)	0.003	**2.0 (1.3 to 3.2)**	**0.003**	**2.4 (1.4 to 3.9)**	**< 0.001**
Miscarriage^b^	15 (37.5%)	14 (23.3%)	0.13	0.5 (0.2 to 1.2)	0.13	**0.2 (0.08 to 0.8)**	**0.02**
No. of previous ETs ≥ 3 (*n* = 272)	(*n* = 143)	(*n* = 129)					
Implantation	60 (42.0%)	61 (47.3%)	0.38	1.2 (0.8 to 2.0)	0.38	1.4 (0.8 to 2.4)	0.20
Clinical pregnancy	32 (22.4%)	44 (34.1%)	0.03	**1.8 (1.1 to 3.1)**	**0.032**	**2.2 (1.2 to 3.8)**	**0.009**
Miscarriage^b^	14 (43.8%)	13 (29.6%)	0.20	0.5 (0.2 to 1.4)	0.2	**0.2 (0.06 to 0.8)**	**0.02**

*Note:* Significant associations are shown in bold.

Abbreviations: CI, confidence interval; HETM, hyaluronan‐enriched transfer medium; OR, odds ratio; RIF, recurrent implantation failure.

^a^ Adjusted for age, BMI, parity, infertility diagnosis, number of embryos transferred and embryo quality.

^b^ Denominator is clinical pregnancies.

^c^ RIF, recurrent implantation failure, defined as patients without pregnancy after two or more previous embryo transfers.

When stratified by the number of previous transfers (Table 3), patients undergoing their first transfer (*n* = 209) showed significant improvement in implantation rates with HETM (adjusted OR: 2.7, 95% CI: 1.4–5.0, *p* = 0.002) and clinical pregnancy rates (adjusted OR: 1.9, 95% CI: 1.0–3.7, *p* = 0.043). For second‐time transfers (*n* = 145), no significant differences were observed between groups. Among 372 cycles with two or more previous failed embryo transfers, the clinical pregnancy rate was significantly higher in the HETM group than in the control group, with an adjusted OR of 2.4 (95% CI: 1.4–3.9; *p* < 0.001). Notably, the miscarriage rate among clinical pregnancies was significantly lower in the HETM group (23.3% vs. 37.5%, adjusted OR: 0.2, 95% CI: 0.08–0.8, *p* = 0.02). Similar results were observed among 272 cycles with three or more previous failed embryo transfers, with an adjusted OR of 2.2 (95% CI: 1.2–3.8; *p* = 0.009) for clinical pregnancy and 0.2 (95% CI: 0.06–0.8; *p* = 0.02) for miscarriage.

### 3.4 Subgroup Analyses

Further subgroup analyses by embryo characteristics are presented in Table 4. Early cleavage‐stage transfers (*n* = 73) showed no significant differences. For blastocyst transfers (*n* = 653), HETM was associated with improved implantation (adjusted OR: 1.6, 95% CI: 1.2–2.3, *p* = 0.004) and clinical pregnancy (adjusted OR: 1.6, 95% CI: 1.1–2.2, *p* = 0.008). Among good‐quality embryo transfers (*n* = 390), HETM was also associated with higher implantation (adjusted OR: 2.0, 95% CI: 1.3–3.1, *p* = 0.002) and clinical pregnancy (adjusted OR: 1.9, 95% CI: 1.2–3.0, *p* = 0.004). In contrast, among poor‐quality embryo transfers (*n* = 336), HETM was not significantly associated with implantation, clinical pregnancy, or miscarriage.

**TABLE 4:** Subgroup analysis for the effect of HETM on clinical outcomes.

Variables	Adjusted OR (95% CI)	*p*
Early cleavage (*n* = 73)^a^		
Implantation	1.3 (0.3 to 6.1)	0.78
Clinical pregnancy	4.7 (0.5 to 44.2)	0.17
Miscarriage^b^	NA	
Blastocyst (*n* = 653)^a^		
Implantation	**1.6 (1.2 to 2.3)**	**0.004**
Clinical pregnancy	**1.6 (1.1 to 2.2)**	**0.008**
Miscarriage^b^	0.6 (0.3 to 1.1)	0.082
Good quality embryos (*n* = 390)^c^, ^d^		
Implantation	**2.0 (1.3 to 3.1)**	**0.002**
Clinical pregnancy	**1.9 (1.2 to 3.0)**	**0.004**
Miscarriage^b^	0.6 (0.2 to 1.4)	0.20
Poor quality embryos (*n* = 336)^c^, ^d^		
Implantation	1.4 (0.9 to 2.3)	0.15
Clinical pregnancy	1.5 (0.9 to 2.6)	0.10
Miscarriage^b^	0.5 (0.2 to 1.5)	0.21

*Note:* Significant associations are shown in bold.

Abbreviations: CI, confidence interval; HETM, hyaluronan‐enriched transfer medium; OR, odds ratio.

^a^ Adjusted for age, BMI, parity, infertility diagnosis, number of embryo transferred and embryo quality.

^b^ Denominator is clinical pregnancies.

^c^ Adjusted for age, BMI, parity, infertility diagnosis, and number of embryo transferred.

^d^ Good quality embryo includes cleavage embryos that were graded as 2 according to the Veeck classification system, and blastocysts that were graded as BB according to the Gardner classification system. Poor‐quality embryos were defined as embryos not meeting the criteria for good‐quality embryos.

## 4 Discussion

This retrospective single‐center study evaluated the effectiveness of HETM in FET cycles by comparing clinical outcomes before and after its implementation. We found that HETM use was associated with significantly higher clinical pregnancy rates than standard transfer medium. Notably, the increased odds of clinical pregnancy were the highest in patients with RIF, and similar findings were observed in the more conservative analysis restricted to those with three or more previous failed embryo transfers. Subgroup analyses suggested benefit in blastocyst transfers and transfers involving good‐quality embryos. These findings suggest that HETM may enhance implantation potential and improve clinical outcomes in FET cycles.

While studies of fresh embryo transfer have demonstrated more consistent benefits of HETM supplementation, the efficacy of HETM in FET cycles remains a subject of ongoing debate, with conflicting results reported across multiple RCTs and observational studies [6, 13]. Yung et al. conducted a large double‐blind RCT involving 550 women undergoing FET and reported no significant improvement in live birth rates with the use of a hyaluronan‐enriched transfer medium (EmbryoGlue; HA concentration, 0.5 mg/mL) compared with the control medium (25.5% vs. 25.8%; relative risk [RR], 0.99; 95% CI, 0.74–1.31) [11]. Of note, their control medium also contained HA (0.125 mg/mL). Similarly, Nishihara et al. randomly allocated participants to HETM (HA concentration 0.5 mg/mL) or a lower‐HA control medium and found no significant differences in clinical pregnancy rates in vitrified–warmed blastocyst transfers (41.8% vs. 46.0%, *p* = 0.40); their study population was characterized by advanced maternal age (36–37 years) and multiple previous transfer attempts (mean 3.6–3.8 cycles) [12]. In contrast, Bhoi et al. reported a significant association between HETM use and improved live birth rates compared with embryo transfer using a conventional non–HA‐enriched transfer medium in a large retrospective cohort of 1298 FET cycles (OR 1.593; 95% CI: 1.170–2.168; *p* = 0.003), particularly in first transfer cycles [9]. This variability across studies may be attributed to differences in patient characteristics, embryo quality, number of previous transfer attempts, and methodological factors, including whether the control medium contained HA.

Given the marked effect observed in the RIF subgroup, our findings add to the accumulating evidence that HETM may be particularly beneficial in patients with RIF. In a randomized study of 314 women aged < 40 years with ≥ 4 previous unsuccessful embryo transfers, Nakagawa et al. allocated patients on the day of embryo transfer to an HETM group or an HA‐free control medium and reported significantly higher pregnancy and implantation rates across fresh day‐3 transfers as well as vitrified–warmed day‐3 transfers performed in either a natural or hormone replacement cycle [4]. More recently, Yan et al. conducted a single‐center retrospective study of 248 women meeting criteria for RIF (defined as no pregnancy after four embryo transfers with good‐quality embryos) and compared EmbryoGlue with standard G‐2 medium containing a lower HA concentration; they reported a significantly higher clinical pregnancy rate (44/111 vs. 38/137, *p* = 0.048) but no statistically significant difference in live birth rate (38/111 vs. 35/137, *p* = 0.136) [7]. In addition, another study suggested that HETM may improve outcomes even when embryo‐related factors are less favorable (morphologically poor euploid blastocyst transfer) [8]. Notably, in our cohort, HETM was also associated with higher implantation and clinical pregnancy rates among patients undergoing their first embryo transfer cycle, and among transfers of good‐quality embryos, suggesting that its potential utility may extend beyond the most challenging populations. Nevertheless, further prospective studies with standardized definitions and careful control of embryo‐ and cycle‐level confounding are needed to confirm these observations and clarify the impact on live birth.

In our study, as in previous studies [9, 14], improvements in implantation and clinical pregnancy rates were observed in blastocyst transfers but not in cleavage‐stage embryo transfers. Several explanations may be considered. First, the sample size for cleavage‐stage transfers (*n* = 73) was much smaller than that for blastocyst transfers (*n* = 653), resulting in limited statistical power to detect between‐group differences. Second, when HETM is used for cleavage‐stage embryos, attachment and implantation may begin earlier and may not be synchronized with the endometrium, potentially leading to implantation failure [15]. Third, conversely, even if HETM is used for cleavage‐stage embryos, the medium may disperse within the uterine cavity and have limited impact by the time implantation occurs. Previous studies have similarly reported no significant differences in implantation and clinical pregnancy rates for cleavage‐stage FET in an RCT [12], whereas others have reported significant improvements [4, 14, 15]. Further studies are needed to clarify the effectiveness of HETM in cleavage‐stage embryo transfer.

A strength of this study is that we evaluated all FET cycles performed in the 2 years before and after implementation of HETM, which allowed assessment of HETM effects across multiple indications and subgroups. In addition, the control medium contained no HA, enabling evaluation of the effect of HA on clinical outcomes, which was not possible in some previous studies, particularly those reporting no significant association with HETM [11, 12]. Because HETM was used for all FET cycles after implementation, selection bias related to indications for HETM use is less likely. However, this study has several limitations. First, the single‐center design may limit generalizability to other populations and clinical settings. Second, because this was a retrospective analysis, it is subject to inherent biases, including residual confounding despite statistical adjustment. Third, we lacked data on some potentially relevant factors, such as endometrial thickness on the day of embryo transfer and embryo aneuploidy status, which may influence both exposure and outcomes. Another limitation relates to the definition of RIF. In this study, RIF was operationally defined as failure to achieve pregnancy after two or more previous embryo transfers; however, this definition may have included age‐related or chance failures. Moreover, because embryo‐quality information for prior failed transfer cycles was unavailable, we could not apply a stricter contemporary definition based on repeated failure after transfer of good‐quality embryos. Nevertheless, additional analysis restricted to patients with three or more previous failed embryo transfers yielded similar results. Finally, because the HETM group had a higher proportion of good‐quality embryos, temporal changes in embryo selection and/or laboratory practice may have contributed to the higher pregnancy rate observed in this group. In addition, add‐on interventions for selected patients with RIF were performed only during the HETM period and not during the control period. Specifically, endometrial scratching‐related procedures were performed in 32 cases and ERA/EMMA/ALICE‐related testing in 5 cases, corresponding to approximately 8.9% and 1.4% of the 358 FET cycles in the HETM period, respectively. Although these frequencies were low, residual confounding related to temporal changes in the management of patients with repeated implantation failure cannot be excluded.

## 5 Conclusion

This single‐center retrospective study demonstrates that HETM use was significantly associated with increased odds for clinical pregnancy in FET cycles, with the most elevated association observed in patients with repeated previous failed embryo transfers. To our knowledge, this study provides real‐world evidence from a historical cohort of routine FET cycles in which HETM was introduced for all transfers and compared with a conventional HA‐free transfer medium, allowing assessment across clinically relevant subgroups such as first‐transfer and RIF cycles. Future prospective RCTs are warranted to confirm these findings and further elucidate which patient subgroups derive the greatest benefit. Based on our results, HETM appears to be a safe and effective intervention that clinicians may consider to optimize FET outcomes.

## Disclosure

Human Rights Statement and Informed Consent: All procedures were performed in accordance with ethical standards of the institutional committees on human experimentation (institutional and national) and the Helsinki Declaration of 1964 and its later amendments. Animal Studies: This article does not contain any studies with animal subjects performed by any of the authors.

## Ethics Statement

The study was approved by the Institutional Review Board of Jichi Medical University Hospital (approval no. 23–205).

## Conflicts of Interest

“Seung Chik, Jwa” is an Editorial Board member of Reproductive Medicine and Biology and a coauthor of this article. To minimize bias, they are excluded from all editorial decision‐making related to the acceptance of this article for publication.

## Data Availability

The data that support the findings of this study are available on request from the corresponding author. The data are not publicly available due to privacy or ethical restrictions.
